# Notices of the Epidemic Constitution of the Summer of 1832, at Cincinnati

**Published:** 1832

**Authors:** Daniel Drake

**Affiliations:** Cincinnati


ART. VI. *Notices of the Epidemic Constitution, of the Summer of* 1832, *at Cincinnati.* By DANIEL DRAKE, M. D.

In the last number of this Journal, I gave some account of the Influenza and Measles which prevailed at Cincinnati, in the winter and spring of the present year. I propose now, at the end of August, to record a notice of the gastro-intestinal irritations, which have constituted the prevalent maladies of the summer. In any other than the present cholera times, such a history might, perhaps, as well not be written; but when the Epidemic is hourly expected, some account of those maladies which would seem to be its precursors, cannot be entirely useless.

The summer just ended, has been remarkable for nothing. It has, in fact, been temperate in all respects—in heat, in rain, in clouds, in wind, and in vegetable productions. The last have been neither more nor less abundant than usual, and in their properties have presented nothing indicative of the least peculiarity in the season. Yet, under this mean term in every quality, the past summer has been uncommonly productive of intestinal affections. These, as usual, began to show themselves about the end of the month of May, and have not yet entirely disappeared. Prevailing to the exclusion of almost every other form of disease, they have constituted a kind of reigning epidemic, and blending themselves with each other, in all their varieties have presented, in fact, but one malady; or at least have evinced themselves the offspring of a common cause. Some patients had vomiting only; others that symptom, with diarrhœa; a much greater number diarrhœa alone; a few real dysentery, and a number, diarrhœa with dysenteric complications; all of which varieties occurred simultaneously, though vomiting, on the whole, was more frequent in June, and dysentery in August.

Within the circle of my own practice, the cholera infantum was not quite as frequent and fatal as usual. Fewer cases than heretofore, were complicated with croup, or terminated in hydrocephalus. Indeed, I saw no instance of the former, and but one of the latter, throughout the whole season. As a general fact, it may be stated, that the warmer the summer, the greater is the prevalence and mortality of cholera infantum, and as the present was not one of our hottest summers, so it was by no means remarkable, for the number and severity of its cases of cholera infantum; nor was the cholera morbus of adults more prevalent or fatal than usual; while the number of dysenteric cases was far less, than I have often known them before. Diarrhœa, however, has been out of all measure, more prevalent than usual, especially among adults. It was not confined to any single class (if such can be said to exist in this country) of the community, but affected the rich and poor, idlers and laborers, the cleanly and the dirty, the old and the young of adult years, nearly in an equal degree. For many weeks this malady, (the *cholerine* of Paris, where, as in other places, it preceded Epidemic Cholera), attracted general attention, and must, I think, have affected nine-tenths of our adult population. Such a prevalence of diarrhœa was certainly never before known in Cincinnati; and suggested to all who knew the history of the dreaded Epidemic, that it was certainly impending. At present, however, this supposed precursor of pestilence has nearly disappeared, and with it the appehension of a more serious visitation.

The following observations on the pathological character of this epidemic constitution, are, perhaps, worth recording.

1. In many cases the individual felt scarcely any indisposition, except a diarrhœa. The morbid action appeared limited to the intestinal canal, and was so mild, that he would eat and drink, and go abroad as usual.

2d. In a great number of cases, more or less fever was awakened, and this, in general, assumed an inflammatory character.

3. Abdominal pain was a common symptom.

4. In almost every case the secretion of bile was nearly or quite suspended. I have not, in any preceding summer, witnessed such an absence of that fluid in the dejections. They were generally thin, light colored, and serous—frequently described by the patient as white. Even when violent vomiting occurred, the matters thrown up, were rarely tinctured with bile.

5. The diarrhœa would cease for several days, and then return, and thus continue to afflict the patient for weeks.

Of the treatment of this epidemic, I shall say but little.

1. Bloodletting and abdominal cupping, were of great service. I saw many cases, which were so decidedly inflammatory, as to call for copious venesection, the omission of which in others, was, I believe, frequently the cause of a protracted disease.

2. Purging was attended with excellent effects. When the intestinal irritation was inflammatory, an oleo-saccharum with mucilage, was soothing and beneficial. In other cases, unattended with inflammatory diathesis, pills, composed of equal parts of calomel, aloes, extract of scammony and compound extract of colocynth, given daily, were productive of the most beneficial results.

3. A pill of calomel and opium, or of those medicines in conjunction with ipecac, administered at night, generally gave great relief, and contributed to the cure. An increase of hepatic and cutaneous secretion, was a common consequence of this practice; and in proportion as these secretions were restored, the intestinal were diminished, and the diarrhœa abated.

4. When the diarrhœa was profuse without fever, the cretaceous mixture with narcotics, was followed by excellent effects; especially, if calomel or the blue pill, was exhibited at night with opium.

5. In all cases, a reduced and demulcent diet was indispensable. Violations in this respect, were often followed by relapses. In some instances, the first attack was, apparently, the consequence of excessive indulgence; in other cases, this cause did not appear to be operative. The prevailing opinion of the physicians and people of the city, was, that fruit and culinary vegetables, would induce the disease; and, in consequence of this sentiment, these articles were used more sparingly this summer than usual. Still, intestinal irritations were general; and hence, I am disposed to believe, that the influence of a vegetable diet in the production of these maladies, is greatly overrated. How, indeed, can succulent and mild acido-saccharine productions, excite inflammation in a surface perpetually accustomed to powerful stimulation by animal food and spices? We must, I think, either believe, that vegetables are not the morbific cause they are supposed to be, or else that the mucous membrane in the diarrhœa lately prevalent, was not inflamed.

So great was the resemblance of this diarrhœa to that which has every where preceded, or constituted the first stage of malignant cholera, that from the moment the physicians of Cincinnati heard of the latter, in the valley of the St. Lawrence, as I have already said, they constantly expected to see it here. Hence every violent case of cholera morbus was contemplated with suspicion, and became a source of popular alarm. The number of such cases was not, I think, as great, or at least, greater, than in common years; but they appeared to be marked by some deviations from their usual character, which gave them an affinity to the pestilential epidemic. Some of these fell under my own observation, and others were communicated to me by my medical friends. It may be well to record a notice of a few of them.

About the 8th of June, I had a female patient affected with obstinate vomiting of glairy matter, great epigastric pain and sinking, extreme reduction of temperature, and repeated cramps and spasms of the muscles of locomotion. She recovered, but had she resided in Montreal instead of Cincinnati, her case would have been reported as one of Epidemic Cholera.

Near the same time, a gentleman was attacked without any known cause, with pain *in epigastrio*, and diarrhœa, attended with considerable fever. The immediate loss of a pint of blood, and the use of a pill of calomel and opium, arrested the disease; but on the same night he awoke with *cramp* in one of his legs, an affection which had never before attacked him in his sleep; and which forcibly turned my mind to the affinity between his malady and the epidemic.

In both of these cases there was defective secretion of bile, which I found it difficult to remove.

For the following facts and cases, I am indebted to my friend Dr. Henry.

"*Cincinnati, July* 9, 1832.

"*Dear Sir,*—I saw several cases of *cholera morbus* during the latter part of the month of May, in one of which, the spasmodic tendency was strongly manifested.

Early in June, I was called to a brick-maker, who had been exposed to the heat of the sun, and of the fire, in burning bricks, and whose habits were somewhat irregular. He had vomiting and purging of a watery description, and violent cramps of the legs and hands.

On the 25th of the same month, I was sent for in great haste, to see a gentleman by the name of Murell, who resided in Clermont county, 33 miles from this city, but who had come to it through a hot and oppressive sun, on the preceding Saturday. He was near 60 years of age, but of temperate habits. On Sunday night before bed time, he had, in rapid succession, several copious watery discharges from the bowels. About 11 o'clock, vomiting commenced, accompanied with violent and painful cramps of the stomach and bowels, and of the muscles of the extremities. He vomited but once after I saw him; but he supposed that he had discharged in all, by vomiting and by stool, at least two gallons of a fluid resembling *muddy water*, which was altogether destitute of odour, or bilious tinge. There was a total want of *urinary secretion*, and this continued until convalescence was well established. His extremities were cold, and the surface generally was bathed in a profuse clammy perspiration; his pulse was small and indistinct, and his tongue was soft, flabby, and covered with a yellow fur. I did not *feel* it so as to ascertain its temperature. He was an intelligent man, and he declared, that although he had frequently had *cholera morbus*, he had never suffered in the same way he did this time.

On the 7th of July, I saw a woman on Front street, and a man on the corner of Front street and Western Row, both of whom had this form of disease. The first had vomiting and purging, great coldness, pain and cramp in the stomach, and in the legs and hands—which were preceded by *numbness* of the extremities, which continued until she was relieved by medicine. The second had abundant watery discharges, violent cramps in the stomach and in the extremities, great coldness of the face, and more especially of the extremities, and copious colliquative sweats. His fingers and toes had the shrivelled and sodden appearance so characteristic of the disease.

On Saturday, July 8th, I was asked to visit a negro man beyond the canal. His name was Reuben Smith, 35 years of age, but of broken down health; having labored under chronic diarrhœa since Aug. last. On the preceding Saturday he had worked unusually hard. He had drunk freely of spruce beer on Friday, and on Saturday he had indulged in copious draughts of iced water. In the afternoon it rained, and the temperature of the air was much reduced. During the night, he had several copious watery discharges: but as he slept alone, and was moreover compelled to go down stairs, the precise character of them could not be ascertained. The next morning about seven o'clock, *vomiting supervened*—but this symptom never attracted much attention, as he vomited only twice, and then discharged merely the food he had eaten the day before, intermixed with a glairy fluid.

I saw him about 10 o'clock, A. M. in company with Dr. Lawrence.

The following are the most prominent symptoms. The circulation in the extremities almost extinct, the pulse at the wrist being so weak, that it could not be distinctly felt: it was somewhat accelerated. His breathing was laborious, and his inspirations short, and seemingly arrested suddenly at a certain stage. His *breath was cold*, and his voice had a low moan, exceedingly unpleasant and distressing; his tongue was soft and parboiled; *it was cold;* so was the whole external surface, and when you touched the extremities, you had the impression of a reduction of temperature, pervading the subcutaneous tissues generally. The whole surface was bathed in a profuse clammy perspiration, and the skin on the fingers and hands, and toes and feet had a sodden, corrugated appearance. His countenance was one of great distress and anxiety, but there did not appear to me to be any thing peculiar in it. He had cramps of the stomach, and abdominal muscles, and of all of the muscles of the upper and lower extremities. I noticed them more especially in the muscles of the fingers, and in the flexor muscles of the thighs. These were aggravated by exertion of any kind. The spasms declined a short time before his death, though his face was convulsed when in the last agonies. He *had no discharge of urine for the last* 12 *hours* of his existence, nor was there any fulness of the bladder. *There was a complete suppression* of the secretion. He had one discharge from the bowels just before I saw him: it was thin, glairy, and white, with *flocculi* of the same color floating in it. He had another about half an hour after I saw him, of the same character; they were destitute of odour or of feculent matter, or of the most remote bilious tinge. The discharge seemed to take place *suddenly*, as if by a spasmodic contraction of the rectum, throwing out its contents, and thus removing all irritation for the time. There was something to my mind very astonishing in his intolerance of external warmth, notwithstanding the deathlike coldness which seemed to be curdling the blood around his heart. He was throughout very thirsty, and very restless; his mind seemed altogether unimpaired. Just before his death, the lips and fingers and toes became *blue*. The blood as it flowed from the vein was dark, thick, tenacious, semicoagulated and running slowly down the arm, from which it fell *guttatim*. He died in about six hours from the time I first saw him, and as nearly as could be ascertained, about 18 hours from the time at which he first complained.

*Dissection*—17 hours after death.

*External aspect*—somewhat emaciated.

*Abdomen*—Omentum. No organic change—veins injected. Stomach and bowels full—mucous coat of the stomach in a state of high inflammation, all around the two orifices, and on the intermediate space along the smaller curvature. Near each orifice there were spots of a sphacelated appearance, of the size of two inches square. The mucous coat in every part, tender, and easily separated from the muscular coat. The stomach was full of the liquids he had drunk, colored by the morbid secretions and by the medicine he had taken. The spleen was in a natural state. The mesenteric glands were greatly enlarged—soft—inflamed, and suppurating in the centre. The mesentery highly injected. The mucous coat of the duodenum, jejunum, and ileum, thicker, tenderer, and more rugous than in health—greatly inflamed in the ileum, and easily abraded—spots of sphacelus, and many patches of a *papillary eruption* of a *white color*, and having minute *black points* in the apex or central part. The colon contained many of these same patches, surmounted with little black drops or points. Throughout the whole course of the primæ viæ, there was found an abundant mucous secretion, of a dark or muddy appearance.

The liver was pale, firm, and almost scirrhous in some parts: it was but little enlarged. The gall bladder was distended with bile, of the usual thick consistence and high color.

The urinary bladder was absolutely empty—the sides lying in perfect contact.

*Thorax.* The lungs free from adhesions or any chronic affection—but little congested, if indeed at all so. The cavæ, and right auricle and ventricle greatly distended with a dark grumous blood—semi-coagulated—and almost destitute of serosity. The left auricle and ventricle so small as to create the impression that they were in a state of hypertrophy, or the others of atrophy.

Respectfully your friend, J. F. Henry, M. D."

For an account of the following case, which occurred in the practice of Dr. Wilie, of this city, I am likewise indebted to Dr. Henry, the consulting physician in the case.

"Mr. Charles Blain, Plumb street, between Front and Columbia, a spare, thin man, had been in delicate health for several years, and for the three weeks preceding, had labored under diarrhœa. He had been exposed as a fireman, the three nights preceding his illness; and as his habits are represented and believed to have been strictly temperate, this exposure was, perhaps, the only exciting cause of his attack. On Friday morning about one o'clock, (July 27th, 1832), he felt a cramp in his feet, legs, and thighs: it was not very annoying, but increased toward day, and advanced to the central parts of the body, the upper extremities becoming simultaneously affected. About nine o'clock, A. M. the spasms became exceedingly severe, at which time he vomited twice, ejecting a thin, glairy fluid, with his food intermixed. His purging consisted of a *thin, frothy, whitish fluid, resembling soap suds.* The accounts I was able to obtain, are not very precise as to the number of the alvine discharges. His brother thinks he had four or five from 9 A. M. until 4 P. M. He himself told me that he had very frequent discharges, but that he supposed they were produced by the medicine. I was called to see him by Dr. Wilie, between 5 and 6 o'clock, P. M. He was then violently affected with cramps of the legs, arms, and muscles of the trunk. His pulse was feeble, beating about 80 strokes in the minute. The temperature of the skin was reduced, though not greatly, below the healthy standard. His veins were flaccid, and when emptied by pressure, it was some time before they regained their half distended size. There was a corrugation of the integuments of his hands and feet. I never saw the Hippocratic face more distinctly marked: the skin seemed drawn tightly over the projecting bones, without muscle or cellular substance interposed. His eyes were retracted; the tunica conjunctiva having underneath it *bloody effusions*—not the *suffusion* which supervenes on *sleep* or *narcotism*, but *blotches* of considerable size, and a *florid* color. He was not decidedly under the influence of opium. I found him awake, and I left him so. He answered my questions promptly, and seemed to understand every thing about him.

Upon enquiry of Dr. Wilie, I found that he had been called to see Mr. Blain between 10 and 11 o'clock, and that he had given him immediately, between 8 and 10 grs. of opium and 40 or 50 grs. of calomel. I am sure the patient was not injured by the opium: I am convinced, that a less quantity of some form of stimulating narcotic would not have been sufficient to control the spasms and carry him through the *cold stage*, or stage of collapse. In addition to the opium, laudanum and æther had been given, and horse-radish *poultices* applied to the upper and lower extremities.

I saw the patient again at 8 o'clock; he was then cold and clammy. I advised hot sand, in bags, to be placed around him; and urged the repetition of internal stimulants, and the application of sinapisms. The *sand* more especially, was wonderfully efficacious in restoring the heat of the surface, and of the extremities.

I shall not dwell on this case. There was no discharge of urine until late on Sunday evening, three days after the attack, when the symptoms of immediate danger had subsided.

Several medical gentlemen who saw him on Sunday, or on Saturday, concluded that he labored under congestion of the brain from opium. But this, in my opinion, was the result of the reaction, or the consecutive fever somewhat augmented by the remedies that were *necessary* to prevent his dieing in the *cold stage.*

I saw Mr. Blain almost two weeks after the attack; he was slowly recovering, and is now I believe quite well."

*Aug.* 20 1832."

The epidemic constitution of which I have given some account, has not been limited to Cinninnati. By gentlemen who have travelled in Kentucky, I learn that it has been extensively prevalent in that State. The same thing is true of Ohio.

The following extract of a letter from Dr. Marble, of Newark, dated August 25, will show its prevalence in that part of the State.

"The diseases of the season have been peculiarly characterized by intestinal irritation. The summer has not been sickly, and neither cholera morbus nor diarrhœa, can be strictly said to have been epidemic; but they have been prominent among the prevailing diseases, as compared with the generality of seasons, when not epidemic.

Cholera morbus was, perhaps, most prevalent during the second and third weeks of July. Within that period I met with several cases in which there were slight cramps: one, the case of a plethoric intemperate man, in which there was much spasmodic affection of the extremities, particularly the calves of the legs and muscles of the abdomen. More recently, I was called to a case in which there was a similar affection of the muscles of the back, which came on immediately after the vomiting and purging, and the pain in the stomach and bowels had been measurably relieved. The patient jirked suddenly and violently, as if springing bodily from the bed, and complained much of numbness of the upper extremities. He had been eating freely of apples and green corn. I took blood from both of these patients—from the latter, largely. Both recovered, as have all others I have seen, by the usual treatment. The disease in this region commonly appears in a mild form.

The diarrhœa of the season has generally been attended with griping in the bowels—frequently with nausea, and has commonly compelled the subjects of it to the use of medical means, and the observance of a strict regimen. In most cases I have been able to trace the exciting cause to the use of the vegetables and fruits of the season, which, as has been very observable, have exerted an unwonted power this summer in producing diseases of this description.

In treating some of the first cases of diarrhœa, I made a free use of siedlitz powders, occupying the interim with anodyne and diaphoretic compositions; but the practice did not do well. I have since advised a single cathartic dose of calomel, following it up for several days with rheubarb and soda, eight or ten grains of each, two or three times a day, conjoined sometimes with a bitter and aromatic tincture. Now and then I have given a large cathartic dose of the rheubarb and soda to begin with, omitting the calomel; and in a few cases, I have had occasion to repeat the calomel. This course has always succeeded.

The intermittent and remittent fevers which have occurred this season, have in many, perhaps, in a majority of instances, made their incursion in the form of a cholera morbus, with severe vomiting and purging. This has commonly, but not always, ceased with the access of the hot stage of the first paroxysm. These cases have generally yielded in a few days, to effectual purging, without the administration of quinine or other tonics. The onset of febrile diseases so generally in this form, is, as far as I know, or can learn, quite a new thing here.

We have not had any dysentery yet—at least, I have not seen a marked case of it, but have seen some cases of neglected diarrhœa tending to that disease.

Very respectfully your ob't. serv't,

D. Marble."

Newark lies about 150 miles east of Cincinnati. I shall transcribe from the letter of an intelligent correspondent, at Steubenville, nearly 100 miles further east, the evidence, that the same atmospheric distemperature has lately prevailed at that place.

"You express a wish to know," says Dr. A. Judkins, "if we have had much *cholera morbus*, or dysentery. In reply, I may inform you, that we have had much more of the former disease this season than is usual, and perhaps, I might say, *less* of the latter.

Complaints of the stomach and bowels have been general; a large majority of these cases did not assume the symptoms of cholera morbus: persons would complain of want of appetite, occasional nausea, frequent pains in the bowels, accompanied generally, though not uniformly with diarrhœa. There was a time, embracing the greater part of June and July, in which I should say, more than one-half the population suffered more or less from some disorder of the stomach or bowels, or both. A want of biliary secretion characterized every case, so far as I know, *whatever might be the amount of indisposition,*

from the most inconsiderable gastric derangement, to the most aggravated case of cholera morbus.

*Cholera morbus* has been more rife this season than I ever knew it in any other. And it must be confessed, that in some cases, to say the least, it has assumed rather a new aspect. In addition to the usual symptoms of that disease, there was *coldness of the skin—perspiration and spasm*, to an extent not hitherto observed. A total absence of bile in most cases, and in two cases in my practice, a total absence of urine for twenty-four hours—and this without enlargement of the bladder—evidencing a suspended secretion.

I think we have had satisfactory evidence of the operation, to a limited degree, of the same cause, which has operated with much fatality in some of the towns and cities to the north and east of us."

*Steubenville*, *Sept.* 3, 1832."

Was the epidemic constitution of the present summer in the West, any thing else than the usual distemperature? I have already stated its characteristics. They certainly differ from those of our ordinary endemic to such a degree, as at least, to constitute it a variety. They assimilate it to, if they do not render it identical with *cholerine*, or the malady which precedes and accompanies Epidemic Cholera. But had this epidemic distemperature a different *cause*, from that which annually reproduces our *endemic* distemperature? If so, what was it? And what *is* the cause of our endemic constitution? Did the former supersede the latter? Or were they capable of combining? If, in reality, but *one* cause exists for both, what agent so far modified it, this summer, as to produce a modified disease? It certainly was not any peculiarity in the sensible meteoration of the atmosphere; for as I have set forth in the beginning of this paper, the summer exhibited no atmospheric peculiarities. I am far from being satisfied with any of the causes usually assigned for our endemic cholera morbus, cholera infantum, and diarrhœa. The theory of intense heat seems plausible, but still intense heat may only develope the real cause; insensible meteoration is but an assumption; and malaria a mere hypothesis.

*Cincinnati*, *Aug.* 31, 1832.

